# Gastric polyps' regression after potassium‐competitive acid blocker cessation

**DOI:** 10.1002/jgf2.552

**Published:** 2022-04-27

**Authors:** Masaya Iwamuro, Hidenori Shiraha, Hiroyuki Okada

**Affiliations:** ^1^ Department of Gastroenterology and Hepatology Okayama University Graduate School of Medicine, Dentistry, and Pharmaceutical Sciences Okayama Japan

**Keywords:** esophagogastroduodenoscopy, fundic gland polyp, hyperplastic polyp

## Abstract

Esophagogastroduodenoscopic examination shows a large, reddish polyp in the gastric cardia. The polyp decreased in size at 10 months and 22 months after cessation of vonoprazan.

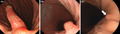

A 78‐year‐old Japanese woman with rheumatoid arthritis and hepatitis B “e” antigen‐negative chronic infection has been taking omeprazole for 13 years, followed by vonoprazan, a potassium‐competitive acid blocker, for 8 months. The patient also received salazosulfapyridine and prednisolone for rheumatoid arthritis. She underwent esophagogastroduodenoscopy screening, which revealed large erythematous polyps (Figures 1A and 2A, arrow) and swollen whitish polyps (Figure 2A, arrowhead). The former was considered hyperplastic polyps, while the latter was fundic gland polyps. Multiple white flat lesions were also observed in the gastric fundus (not shown). Vonoprazan suspectedly promoted gastric polyp growth. Thus, the antacid was switched from vonoprazan to famotidine, a histamine‐2 blocker. Based on the urea breath test performed 2 months after vonoprazan cessation, she was negative for *Helicobacter pylori* infection. IgG antibodies to *H. pylori* were also negative. Esophagogastroduodenoscopy performed 10 months after vonoprazan cessation showed regression of both hyperplastic (Figures 1B and 2B, arrow) and fundic gland polyps (Figure 2B, arrowhead). The sizes of the hyperplastic polyps further decreased 22 months after vonoprazan cessation (Figures 1C and 2C, arrows), while the fundic gland polyps remained unchanged (Figure 2C, arrowhead).

**FIGURE 1:**
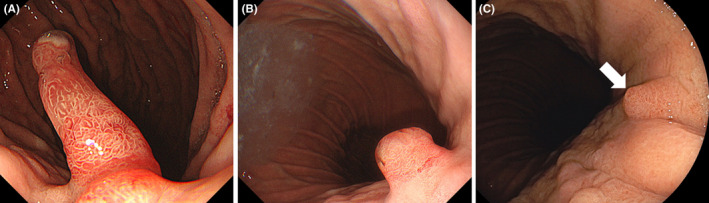
Endoscopic images of a gastric hyperplastic polyp Esophagogastroduodenoscopic examination shows a large, reddish polyp in the gastric cardia (A). The polyp decreased in size at 10 months (B) and 22 months (C) after cessation of vonoprazan

**FIGURE 2:**
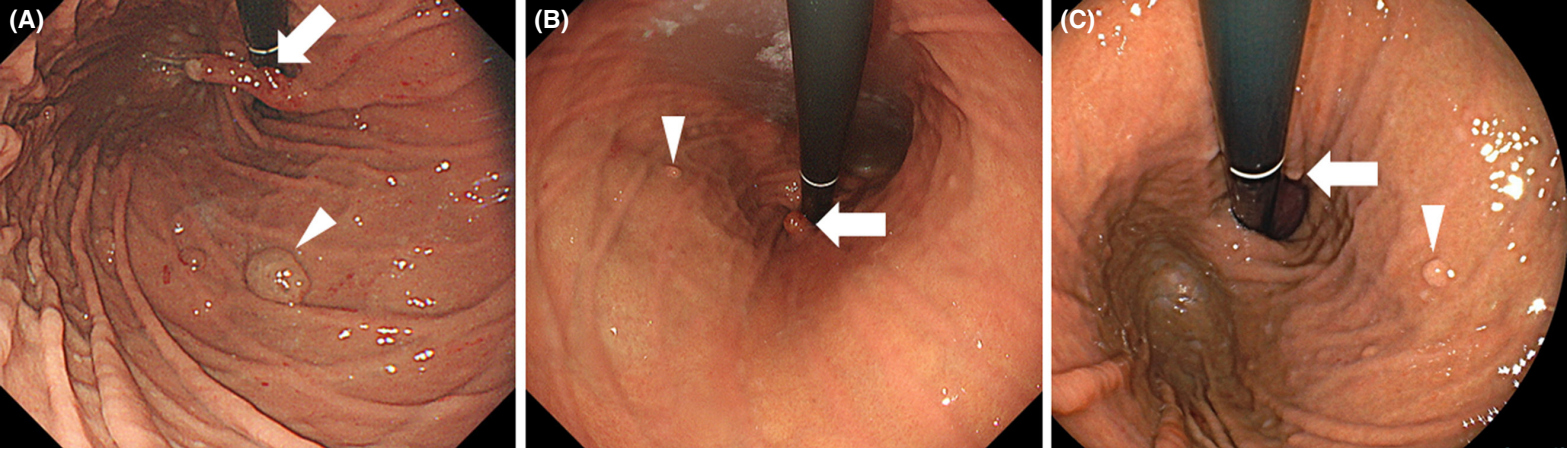
Endoscopic images of a gastric hyperplastic polyp and fundic gland polyps. A gastric hyperplastic polyp (A, arrow, same polyp in Figure 1) and fundic gland polyps (A, arrowhead) are seen. Both polyps regressed at 10 months (B) and 22 months (C) after cessation of vonoprazan

Intervention is warranted in patients with large gastric polyps that have a potential risk of bleeding. Gastric hyperplastic polyps are associated with chronic gastritis induced by *H. pylori* infection ^1^. Since *H. pylori* eradication causes the disappearance of gastric hyperplastic polyps, *H. pylori* eradication is the first‐line treatment for gastric hyperplastic polyps. However, the patient was negative for *H. pylori* infection.

Proton pump inhibitors and vonoprazan have been widely used to treat acid‐related disorders. These drugs are safe, but long‐term use induces histopathological alterations in the stomach, including parietal cell protrusion, cystic dilation of fundic glands, and foveolar epithelial hyperplasia ^2^. These pathological changes lead to hyperplastic and fundic gland polyps in the stomach ^3^. Few studies have reported the disappearance of gastric hyperplastic polyps after the discontinuation of proton pump inhibitors ^4^, ^5^.

In this patient, the large hyperplastic polyps and swollen fundic gland polyps in the stomach prompted vonoprazan discontinuation, resulting in significant gastric polyps regression. These results suggested that vonoprazan use was associated with the development and enlargement of the gastric hyperplastic and fundic gland polyps. Moreover, these polyps may regress or disappear after vonoprazan cessation.

In conclusion, the present case exhibited two types of gastric polyps, namely hyperplastic and fundic gland polyps. Both types regressed after vonoprazan cessation. Thus, vonoprazan discontinuation should be considered in patients with large hyperplastic or fundic gland polyps in the stomach.

## CONFLICT OF INTEREST

H. Okada received an honorarium from AstraZeneca Public Limited Company, Daiichi Sankyo Company, Takeda Pharmaceutical Company, and Otsuka Pharmaceutical Company; research funds from Otsuka Pharmaceutical Company; and donations from Nippon Kayaku Company and EA Pharma Company.

## CONSENT

Written informed consent was obtained from the patient for publication of this case report and accompanying images.
